# Autoimmunity against laminin 332

**DOI:** 10.3389/fimmu.2023.1250115

**Published:** 2023-08-10

**Authors:** Sabrina Patzelt, Enno Schmidt

**Affiliations:** ^1^ Lübeck Institute of Experimental Dermatology (LIED), University of Lübeck, Lübeck, Germany; ^2^ Department of Dermatology, University of Lübeck, Lübeck, Germany

**Keywords:** autoimmunity, diagnosis, malignancy, immunofluorescence, guidelines, BP180, type VII collagen, laminin 332

## Abstract

Laminin 332 is a heterotrimeric structural protein of the basal membrane zone (BMZ) of the skin and adjacent mucosal tissues. The importance of laminin 332 for the structural integrity of the BMZ is demonstrated by mutations in any of the three genes encoding for its three chains causing variants of junctional epidermolysis bullosa. Autoimmunity against laminin 332 is observed in mucous membrane pemphigoid (MMP) and in the rare patients with orf-induced pemphigoid. MMP is an autoimmune blistering disease with predominant mucosal manifestations and autoantibodies against the BMZ of the skin and orifice-close mucous membranes. The main autoantigens of MMP are type XVII collagen (BP180) and laminin 332 targeted in about 80% and 10-20% of patients, respectively. An increasing number of studies has highlighted the association of anti-laminin 332 MMP and malignancies that can be revealed in about a quarter of these patients. This data has led to the recommendation of current guidelines to assay for anti-laminin 332 reactivity in all MMP patients. The present review focuses on anti-laminin 332 MMP describing clinical features, its pathophysiology, and detection of serum anti-laminin 332 IgG. In addition, the available data about the occurrence of malignancies in anti-laminin 332 MMP, the underlying tumor entities, and its biology are detailed.

## Introduction

Laminin 332 is a heterotrimer and essential structural protein of the basal membrane zone (BMZ) of the skin, adjacent mucosal tissues including the mouth, pharynx, larynx, trachea, esophagus but also kidney, lung, and small intestine (1). The importance of laminin 332 for the structural integrity of the BMZ is demonstrated by mutations in any of the three genes *LAMA3*, *LAMB3* and *LAMC2*, that cause a variant of junctional epidermolysis bullosa (2, 3). Autoimmunity against laminin 332 is observed in the autoimmune blistering disease mucous membrane pemphigoid (MMP) and in the very rare patients with orf-induced pemphigoid (4, 5). Furthermore, autoantibodies against laminin 332 have been described in individual patients with bullous pemphigoid, anti-p200 pemphigoid, and epidermolysis bullosa acquisita in addition to the disease-typical autoantibodies against, i.e. BP180/type XVII collagen, p200 protein, and type VII collagen, respectively (6–11). The present review focuses on anti-laminin 332 MMP summarizing clinical features, its pathophysiology, and detection of serum anti-laminin 332 IgG. In addition, the current data about the association between anti-laminin 332 MMP and malignancies are highlighted.

The current review is dedicated to the late Detlef Zillikens, director and chair of the Department of Dermatology, University of Lübeck, Germany. Detlef Zillikens has been one of the leading experts on autoimmune blistering diseases. With an enormous workload and his friendly, optimistic, supportive, and caring nature he has established in Lübeck one of the world largest research hubs for these disorders. As one of his first students in 1993, close collaborator, mentee, and friend, E.S. owes him the greatest thanks for constant support, motivation, and fruitful discussions. S.P. got to know Detlef Zillikens in 2016 when starting her PhD thesis and owes him the greatest respect and thanks for his support of a young scientist and incessantly enjoyment of research. He was able to close the gap between science and clinic due to his dedication for both disciplines and his view of the entire picture. Both authors will strive to continue Detlef’s work and guard the best memories of him.

## Laminin 332

Laminins are cross- or T-shaped heterotrimers of an α, β and γ chain with three short arms (single chains) and one long arm formed by all three chains (12). Laminins are integral proteins of the BMZ of the skin and surface-close mucosal tissues. Here, they are essential components of the anchoring filaments connecting the hemidesmosome with type VII collagen (13). Their physiological functions include adhesion of the epidermis to the dermis and epithelium to the lamina propria, respectively, cell migration and, cell signaling (12).

Laminin 332, previously termed laminin 5, epiligrin, nicein, and kalinin is composed of the α3, β3 and γ2 chains and expressed in the BMZ of e.g. oral mucosa, conjunctiva, skin, kidney, lung, and small intestine (1). In the skin, laminin 332 is synthesized by keratinocytes as a 460 kDa precursor protein that is extracellularly cleaved by proteases. As such, the α3 chain (190-200 kDa) is processed into a 165 kDa fragment, the 155 kDa γ2 chain in a 105 kDa fragment, while the 140 kDa β3 chain remains uncleaved (13). Laminin 332 interacts with BP180 (type XVII collagen), the NC-1 domain of type VII collagen (14, 15), with α3β1, α6β4, and α6β1 integrin as well as with syndecan-1 and syndecan-4 (16, 17).

## Mucous membrane pemphigoid

**Figure 1:**
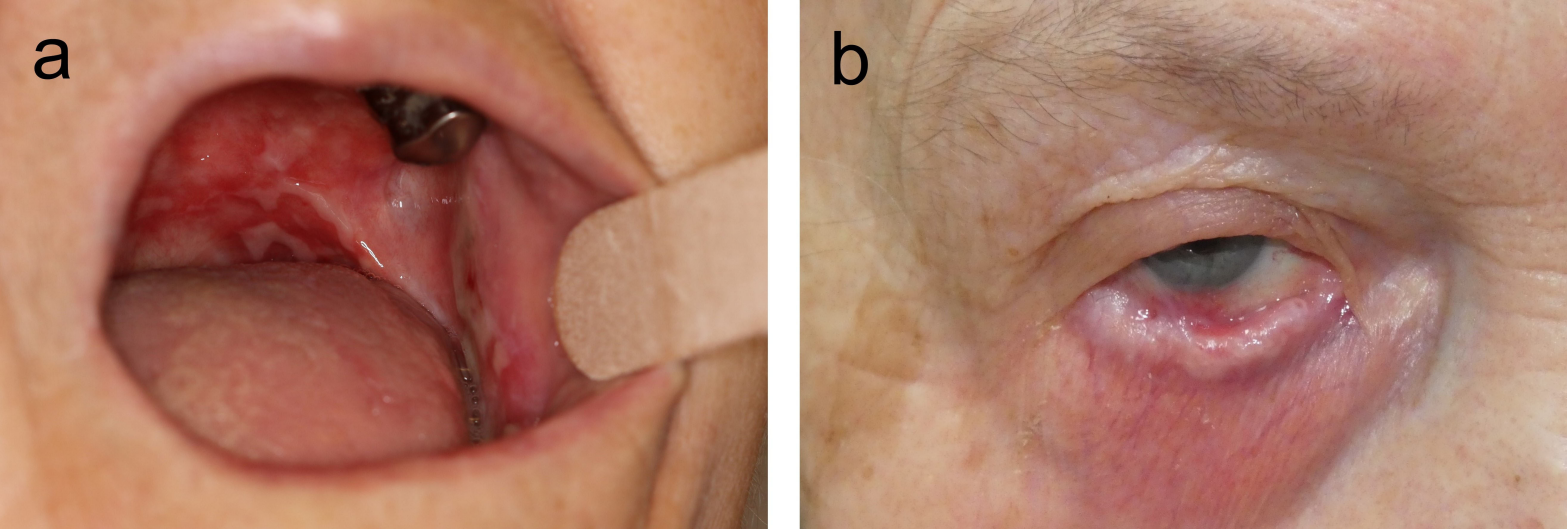
Clinical manifestations of mucous membrane pemphigoid. Extensive oral lesions in an 83-year-old female patient **(A)** and symblephara and shortening of the inferior fornix in a 72-year-old female **(B)**.

MMP is a clinically and immunopathologically heterogeneous disease defined as pemphigoid disorder with prevailing involvement of orifice-close mucosal tissues (18). As a pemphigoid disorder, MMP is characterized by autoantibodies that bind to the BMZ of the skin and/or mucosa (19, 20). Clinical heterogenicity is reflected by the involvement of different mucosal sites, most frequently the mouth (in about three quarters of patients) and conjunctivae (in about 50-65% of patients) followed by nasopharynx and genitalia, and more rarely, larynx, esophagus, and trachea. In about a quarter of patients, in addition to mucosal manifestations, skin lesions are present (**Figure 1**) (21, 22). The high disease burden of MMP is due to frequently painful oral and genital lesions, life-threatening complications such as airway obstruction and esophageal strictures, conjunctival disease leading to vision impairment and finally, blindness, and the association with a malignancy in about a quarter of patients with anti-laminin 332 reactivity (22).

Immunopathological heterogenicity stems from the different target antigens and the autoantibody isotype. While in most MMP patients, autoantibodies belong predominantly to the IgG isotype, the majority of patients also reveal IgA autoantibodies, and in some, the autoantibody response is restricted to IgA (22–24). BP180 (type XVII collagen) as main target antigen in MMP is recognized by about 70-80% of patients followed by laminin 332 in 10-20% of patients. In less than 5% of MMP patients, type VII collagen is recognized. Reactivity against BP230, that can be found in 10-30% of cases, is nearly always accompanied by autoantibodies against one of the three other target antigens (21, 24). In some MMP patients, autoantibodies against α6β4 integrin have been described (25–28). The relevance of these α6β4 integrin-specific antibodies in MMP is, however disputed (24, 29). Patients with mostly mucosal manifestation and predominant IgA reactivity, that previously may have been classified as linear IgA disease, and those with autoantibodies against type VII collagen previously diagnosed as epidermolysis bullosa acquisita, are now regarded within the spectrum of MMP (21).

Few data about the frequency of MMP are available. With an incidence between 1.3 and 2.0/million/year in France and Germany, respectively, and a prevalence of 24.6 patients/million in Germany, MMP is certainly a rare disease (30–33). MMP arises independently of ethnicity and geographical region, mainly affects individuals in the 7^th^ and 8^th^ decennium, and appears to be more frequent in females (22).

**Figure 2:**
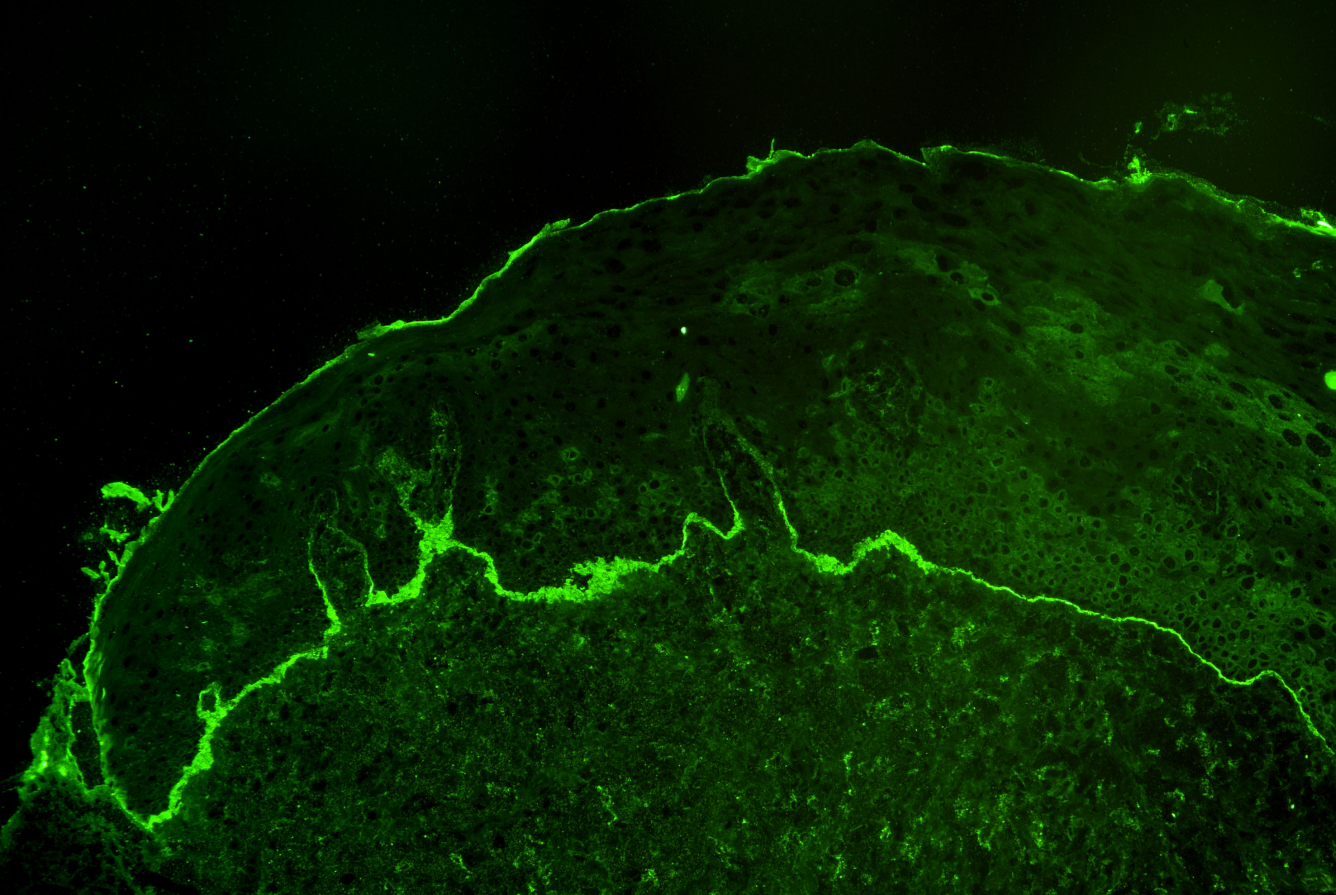
Linear deposits of complement C3 at the basement membrane zone by direct immunofluorescence microscopy of a perilesional biopsy in a patient with mucous membrane pemphigoid.

Diagnosis of MMP, like in all autoimmune blistering diseases, is grounded on three pillars; clinical manifestations, direct immunofluorescence (IF) microscopy, and serology (20, 24). The clinical prerequisite is predominant mucosal involvement. Direct IF reveals linear deposits of IgG, IgA, and or C3 at the cutaneous or mucosal BMZ in a non-lesional biopsy (**Figure 2**). Since the initial biopsy only provides a sensitivity of 50-70% depending on the biopsy site, current guidelines recommend to repeat the biopsy for direct IF at least once after an initially negative result (24, 29).

Detecting of circulating autoantibodies against the above-mentioned antigens is complex, mainly based on in-house assays, and reviewed elsewhere (22, 24). The detection of anti-laminin 332 IgG is detailed below.

Treatment of MMP is greatly hampered by the lack of randomized controlled studies. National and international guidelines propose treatment regimens (24, 34–37). The S3 European guidelines included a systematic literature review and recommend dapsone, methotrexate, tetracycline, and topical corticosteroids as first line treatment for mild and moderate MMP. For severe MMP, dapsone plus cyclophosphamide and/or oral corticosteroids are suggested and, if not successful, dapsone plus rituximab followed by latter two drugs combined with high-dose intravenous immunoglobulin (24). A slightly different step-ladder approach was published in the recent German S2k guideline (29).

## Anti-laminin 332 mucous membrane pemphigoid

In 1992, laminin 332 has been described as a target antigen in MMP by Kim Yancey and co-workers (4). Since then, numerous case reports and case series have reported IgG serum autoantibodies against this protein. It was only in 2019, when a highly standardized and specific assay for serum anti-laminin 332 IgG became widely available (38).

### Clinical appearance of anti-laminin 332 mucous membrane pemphigoid

A patient with anti-laminin 332 MMP can clinically not be differentiated from a MMP patient with autoimmunity against BP180 or type VII collagen. In a systematic review of published cases and cohorts, Amber et al. reported significantly more pharyngo-laryngeal and oro-pharyngo-laryngeal involvement in MMP patients with reactivity against laminin 332 (39). In the so far largest study with 133 anti-laminin 332 MMP patients from Kurume, Japan, the oral cavity was the by far most frequently affected mucosal site (in 89% of patients) followed by conjunctivae (in 43%), pharynx (in 19%), larynx (15%), genital mucosa (in 11%), nasal mucosa (in 6%), and esophagus (in 3%) (40). Compared with MMP patients independent of the target antigen as recently reported in 154 MMP patient and as reviewed by Du et al., nasal lesions appear to occur less frequently in anti-laminin 332 MMP compared to 20-40% in all MMP patients, while oral lesions may be slightly more prominent (in 80-85% of all patients) (22, 41). These differences have, however, not been systematically evaluated and may also be related to the different ethnicity or other so far unrecognized factors.

Recently, a significant association of laminin 332-reactive MMP with male sex was reported (41). The most striking and clinically relevant feature that differentiates anti-laminin 332 MMP from MMP with other autoantibody reactivities, the association with malignancies in about a quarter of patients, is detailed below.

### Detection of anti-laminin 332 reactivity

Several methods have been applied to detect anti-laminin 332 reactivity in skin and mucosal biopsies as well as in serum. Direct and indirect immunogold electron microscopy show deposits of immunoreactants at the lamina lucida/lamina densa interface of the BMZ in anti-laminin 332 MMP. In patients with autoantibodies against BP180 or type VII collagen, immunoreactants label the lamina lucida or the subbasal lamina-anchoring fibril zone, respectively (4, 42–45). Direct immunogold electron microscopy requires, however, fresh biopsy material that needs to be processed within hours and is only performed in few centers worldwide (46).

For the detection of serum autoantibodies against laminin 332, indirect immunogold electron microscopy is unpractical and as such, several in-house assays have been described including (i) immunoprecipitation of radiolabeled keratinocytes that was also applied in the original report of anti-laminin 332 IgG in MMP (4, 47), (ii) immunoblotting with various substrates such as (a) conditioned media of cultured SCC-25 cells (48), (b) cultured primary human keratinocytes (47, 49), (c) cultured HaCaT keratinocytes (50), (d) cultured A-431 human epidermoid carcinoma cells (50), (e) extracts of human epidermal sheets (50), (f) extracellular matrix of cultured human keratinocytes (45, 50), (g) extract of human placental amnion (51), (h) recombinant fragments of the α3 chain (52), (i) human laminin 332 purified from cultured human keratinocytes (53), (j) primary human oral mucosal keratinocytes (54), and (k) immortalized human oral mucosal keratinocytes (54), and (iii) ELISA. When immunoprecipitation was compared to immunoblotting with five different substrates, i.e. (b-f), immunoprecipitation was identified as the most sensitive method followed by Western blotting with extracellular matrix of cultured human keratinocytes (II f) (50).

For ELISA, purified laminin 332 from conditioned medium of cultured SCC-25 cells (47, 55, 56), recombinant laminin 332 (57), laminin 332 purified from supernatant of cultured primary human keratinocytes (57), or extracellular matrix of cultured HaCaT keratinocytes were used (57). In particular the ELISA employing purified laminin 332 from conditioned medium of cultured SCC-25 cells has subsequently revealed conflicting results. Bekou et al. reported anti-laminin 332 IgG in 40% of bullous pemphigoid sera, although anti-laminin 332 reactivity is not present in latter patients (38, 55, 58, 59). Bernard et al. described serum anti-laminin 332 IgG in 31 of 154 MMP patients; when 19 of the 31 laminin 332-reactive sera were retested, anti-laminin 332 reactivity was only confirmed in 4 of the 19 sera (60).

In sera with reactivity against the cutaneous BMZ by indirect IF microscopy on human skin, indirect IF on laminin 332-deficient skin from patients with junctional epidermolysis bullosa (being unreactive on latter substrate) as well as the fluorescence overlay antigen mapping on human salt-split skin are elegant methods to determine autoantibodies against laminin 332 (61). Another test based on indirect IF, the so-called footprint assay, demonstrated that anti-laminin 332 serum IgG can be detected in the extracellular matrix of cultured primary keratinocytes after removal of the cells from the glass coverslips. Here, the extracellular matrix of the removed individual keratinocytes appear as traces or “footprints” that can be visualized by anti-laminin 332 antibodies followed by FITC labelling (59).

**Figure 3:**
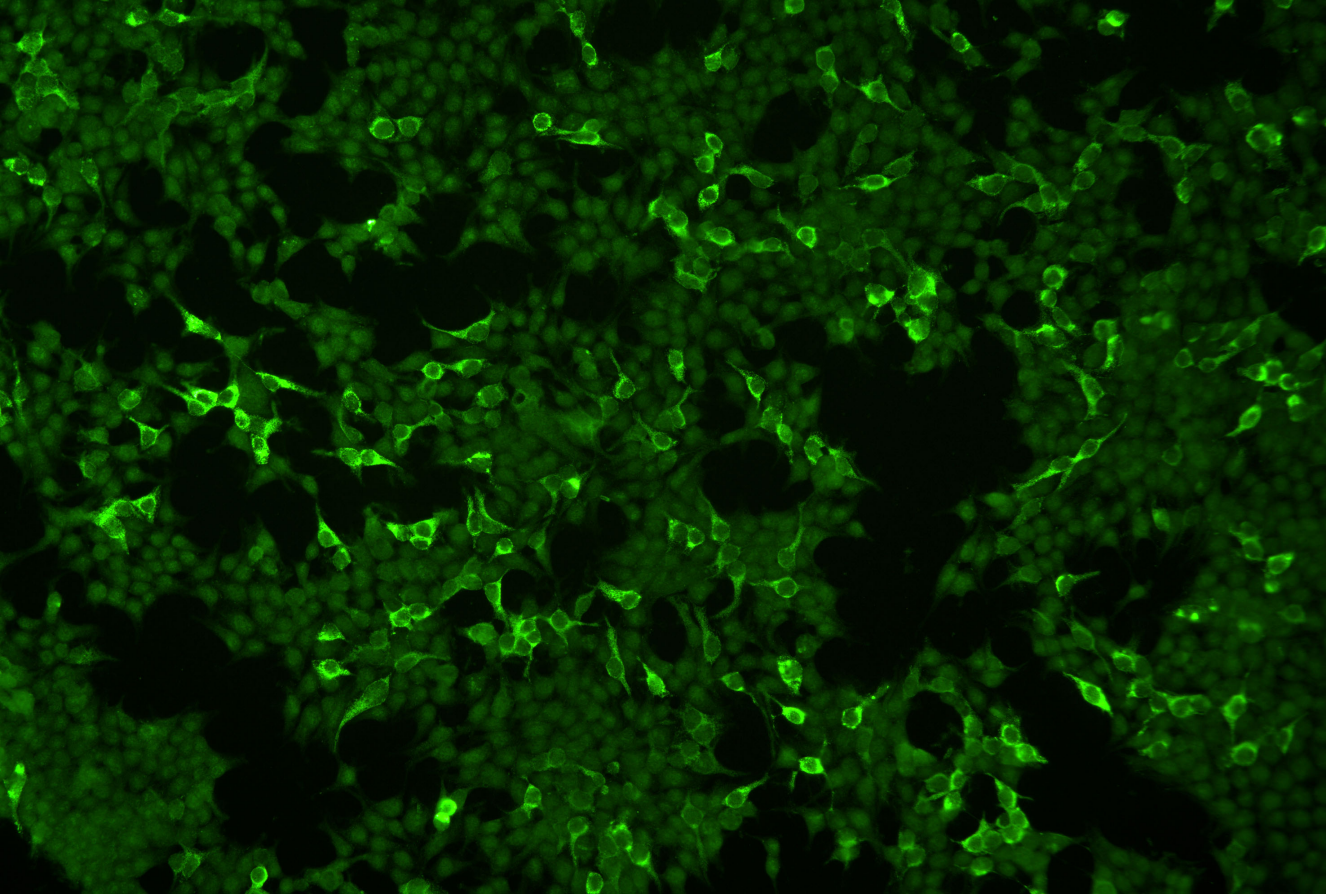
Indirect immunofluorescence microscopy of HEK293 cells that recombinantly express laminin 332 on the cells surface employing the Biochip™ technology. A serum of a patient with mucous membrane pemphigoid labels laminin 332-expressing cells. Non-transfected cells serve as internal negative control.

A breakthrough was achieved by Goletz et al. who described an indirect IF test based on the HEK293 cells that recombinantly express the laminin 332 trimer on their cell surface (**Figure 3**). As negative control, HEK293 cells transfected with an empty vector are used. These cells are applied using the BIOCHIP^®^ mosaic technology, i.e. several substrates are placed together in a single incubation field of a laboratory slide (62–65). When in an international multicenter study, 93 anti-laminin 332 MMP patient sera and 315 sera from other autoimmune blistering diseases including 153 sera from anti-laminin 332 negative MMP patients, non-inflammatory dermatoses, and heathy blood donors were probed, a sensitivity of 84% and a specificity of 99.6% were observed (38). This assay has subsequently been validated by other groups (66, 67). When the BIOCHIP^®^ technology-based assay has recently been compared with the footprint assay using 54 anti-laminin 332 MMP sera and together 50 sera from patients with pemphigus vulgaris and healthy blood donors, both assays revealed a specificity of 100% with a slightly higher sensitivity of the footprint assay (100% versus 96.3%) (60). When 35 sera of originally laminin 332-unreactive sera were subjected to both IF tests, 3 were reactive in the BIOCHIP^®^ assay and 7 in the footprint assay. These data show that the footprint test may be more sensitive, whereas the advantage of the BIOCHIP^®^ assay is its high standardization and wide availability (60).

Reactivity against the different laminin chains varied considerably between studies. In 113 Japanese patients with anti-laminin 332 MMP, the γ2 chain was most frequently recognized (in 58% of patients) followed by α3 and β3 targeted in 49% and 36% of patients, respectively (40). In contrast, Goletz et al., using the BIOCHIP^®^ technology-based IF assay in an international multicenter study with 93 sera, reported IgG4 reactivities against the α3, β3, and γ2 in 43%, 41%, and 13% of patients (38). These discrepancies maybe most likely due to the different study populations or detection methods.

In individual MMP patients, IgA and IgE antibodies against laminin 332 have also been reported (68, 69).

**Figure 4:**
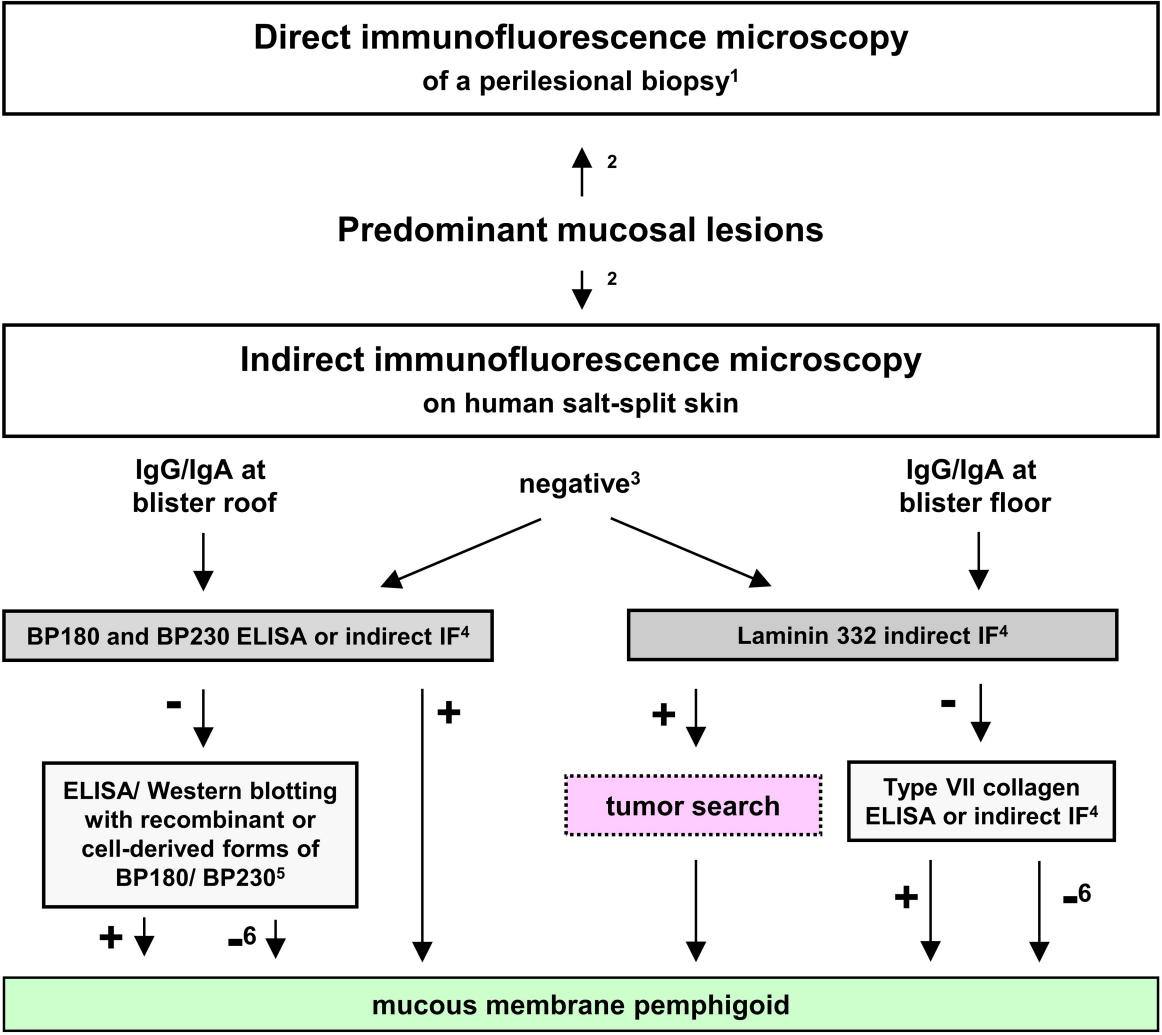
Proposed diagnostic algorithm for mucous membrane pemphigoid (MMP). Adopted from (22, 24, 70). ^1^in the oral cavity, a non-lesional biopsy is equally sensitive compared to a perilesional; ^2^recommended to be performed in parallel; ^3^in 30-50% of MMP sera; ^4^commercially available (for IgG); ^5^only available in specialized laboratories as in-house assays; ^6^only with positive direct and/or indirect IF microscopy.

Since anti-laminin 332 MMP is associated with a malignancy in about a quarter of patients as detailed below, national and international guidelines recommend the detection of anti-laminin 332 serum IgG in all patients that show dermal binding by indirect IF on human salt-split skin or were unreactive in this assay (24, 29). A suggested diagnostic pathway for anti-laminin 332 MMP is depicted in **Figure 4**.

### Association of anti-laminin 332 mucous membrane pemphigoid with malignancies

Gibson et al., have observed the first patient with anti-laminin 332 MMP and a malignancy, a lung carcinoma, in 1997 followed by Leverkus et al., who reported solid malignancies in 2 of 5 MMP patients with serum reactivity against laminin 332 (71, 72). The association was first noted by Egan et al. who described malignancies in 10 of 35 (29%) anti-laminin 332 MMP patients (73). When all nine subsequent studies with more than three anti-laminin 332 MMP patients were evaluated, a clear association with malignancies was evident. In fact, 57 of 253 (23%) patients with anti-laminin 332 reactivity had a malignancy (**Table 1**). These data align well with a recent review in which Shi et al., retrieved 344 reported cases of anti-laminin 332 MMP from the literature, of whom in 75 (22%), a malignancy was described. Van Beek et al. calculated the risk for malignant neoplasms in anti-laminin 332 MMP to be 6.8-fold higher compared to the general population (41).

**Table 1:** Association of anti-laminin 332 MMP with malignancies^1^ .

Study	Year of publication	Origin of patients	No. of patients	No. of patients with malignancy
Leverkus et al. (72)	1999	Germany	5	2 (40%)
Egan et al. (73)	2001	USA	15	5 (29%)
Matsushima et al. (74)^2^	2004	Japan	16	5 (31%)
Terra et al. (47)	2011	Netherlands	10	2 (20%)
Bernard et al. (56) Goletz et al., (60)	2013	France	[31] 4	[2 (6%)]^3^ 2 (50%)^3^
Hayakawa et al., (75)	2014	Japan	4	2 (50%)
Goletz et al. (38)	2019	Germany, Japan, France, Italy, USA	53^4^	13 (25%)
Li et al., (76)	2021	Japan	[55]^4,5^	[8 (14%)]^5^
Qian et al. (40)	2021	Japan	133^4^	22 (17%)
van Beek et al., (41)	2021	Germany	13^4^	4 (31%)
**Total**			**253**	**57 (23%)**

^1^only studies with more than 3 patients are indicated; ^2^review of Japanese cases; ^3^when 17 of the 31 reported sera were re-analyzed by the Biochip^®^-based indirect IF assay only 4 reacted with laminin 332. Of these 4 sera, 2 had a malignancy (60). As such, here, only latter data were included; ^4^ some patients may have also been included in other studies listed here; 5 data of this study were not included in the total numbers since all patients also appeared in the study of the same group by Qian et al. (40). Total numbers are shown in bold.

In the recent review by Shi et al., the most frequent tumor in 84 malignancy-associated anti-laminin 332 MMP patients retrieved from the literature, were lung carcinomas (in 23% of patients) followed by gastric (in 17%), uterine (in 13%), pancreatic (8%), colon (8%), ovary (7%), prostate (5%), and thyroid carcinoma (5%) (77). No relation between the recognized laminin chain and the tumor entity was found (77). Of the 12 malignancy-associated anti-laminin 332 MMP patients reported by Goletz et al., 3 (25%) had a lung and 2 (17%) a uterine/cervix carcinoma compatible with the data reported by Shi et al., while 2 (17%) revealed a urothel carcinoma and none has a gastric malignancy (38, 77). These data suggest that in anti-laminin 332 MMP, solid malignancies predominate with lung and uterine/cervix cancers being among the most prevalent entities, while the distribution of other solid malignancies may also depend on the population.

Interestingly, in patients with serum reactivity against α6β4 integrin, no higher rate of malignancies was found alike in MMP patients in general irrespective of the target antigen (78–80).

The exact reason for the association of ani-laminin 332 reactivity and solid cancers has not been fully elucidated yet. It is well known that laminin 332 is relevant for tumor proliferation and migration (81–83). Some solid tumors may produce excessive amounts of laminin 332 and an imbalance of extracellular matrix proteins including laminin 332 was shown to promote tumor cell migration via the Pi3-akt pathway as well as the differentiation of tumor-associated fibroblasts and tumor angiogenesis (84–86). As such, it may be hypothesized that an imbalance in laminin 332 expression during carcinogenesis induces an autoimmune response that leads to laminin 332-specific autoimmunity including anti-laminin 332 antibodies (87–89). This view is supported by the observation that MMP can regress after excision of the tumor (87, 90, 91).

### Pathophysiology of anti-laminin 332 pemphigoid

**Figure 5:**
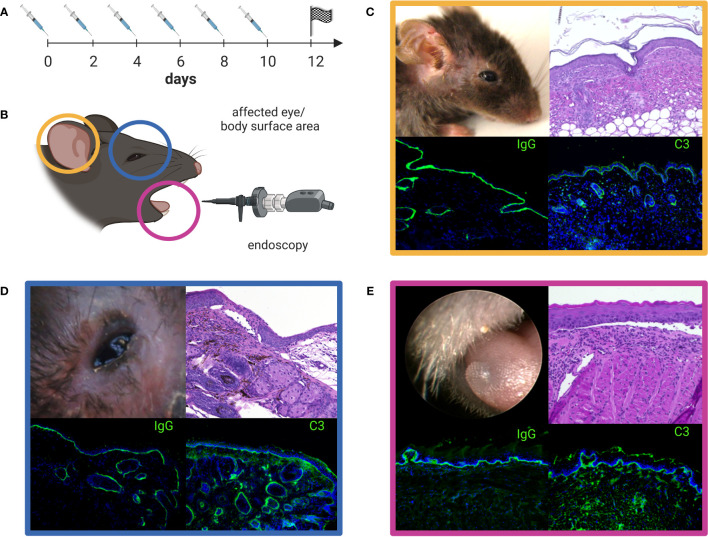
Anti-laminin α3 mucous membrane pemphigoid (MMP) mouse model. Rabbit anti-murine laminin α3 IgG is injected subcutaneously (s.c.) into adult C57Bl/6 mice every other day over a time period of 10 days **(A)**. The clinical manifestation of the mouse model seen on experimental day 12 can be quantified by the use of a validated scoring system comprising the affected body surface area (yellow, **C**), the affected eye-area (blue, **D**), and the severity of oral lesions as examined by endoscopy (pink, **E**) **(B)**. The color-framed boxes **(C-E)** show the clinical presentation (upper left panel), H&E stained lesional histopathology with an inflammatory infiltrate and split formation of the dermal-epidermal/epithelial junction (upper right panel) and, linear deposits of IgG (lower left panel) and C3 (lower right panel) along the basal membrane zone by direct immunofluorescence microscopy. Lesions, crusts and erosions of the skin are mostly restricted to the head, neck, and upper back of mice **(C)**. The image was created with BioRender.com.

Preliminary evidence for the pathogenic relevance of anti-laminin 332 IgG stems from the intraindividual correlation of anti-laminin 332 IgG serum levels with disease activity (38). Apart from *in-vitro* organ culture models of MMP employing normal human conjunctiva (25, 92–94), two mouse models of anti-laminin 332 MMP have been developed. One model reflects the inflammatory-poor variant of MMP and lesions develop independently of complement activation and the infiltration of inflammatory cells in the tissues, while the other model shows, oral, conjunctival, and skin lesions with inflammatory infiltrates and requires the involvement of the Fcγ-receptor and activation of C5aR1 (95–97). In latter model, dapsone has recently been shown to be effective supporting the notion that this model recapitulates important features of the human disease (98). Because most recent publications used the latter mouse model, a detailed description is depicted in **Figure 5**. In line with previous findings, methylprednisolone as another first-line therapy for MMP, was also able to reduce the severity of skin, although not oral lesions in this mouse model. In this study, Ghorbanalipoor et al. also showed that parsaclisib, a selective inhibitor of phosphoinositide 3-kinase delta (PI3Kδ) significantly reduced skin and oral mucosal lesions (99, 100). With regard to the characteristic symptom of scarring, typically occurring at the eyes of anti-laminin 332 MMP patients, this mouse model may also be suitable to unravel signaling pathways that contributes to this specific immunopathogenesis. Biopsies of the palpebral conjunctiva and the skin collected 28 days after the initiation of this model revealed highly condensed collagen fibrils in picro-sirius red staining and trichrome histological staining. In addition, biochemical analysis provided results on altered collagen-cross-linking signaling pathways in these tissues that are associated with fibrosis (101). Furthermore, the previously published upregulation of aldehyde dehydrogenase (ALDH1) in conjunctiva and in fibroblasts isolated from MMP patients with severe eye involvement, could be verified by transcriptome analysis of perilesional skin from this model (102). The inhibition of ALDH1 by disulfiram decreased disease severity in a mouse model for allergic eye disease (102). However, disulfiram was not effective in the anti-laminin 332 mouse model. Here, the same dosage and application of disulfiram was not able to reduce the severity of the conjunctival lesions (101).

*In-vitro* models specific for anti-laminin 332 pemphigoid are rare. Recently Bao et al. published results about anti-laminin 332 MMP patient antibodies that were sufficient to release inflammatory mediators upon binding to keratinocytes without the presence of inflammatory cells and as such without the usage of Fc-receptors. Thus, arising the question whether blistering may be a consequence of just the binding of the anti-laminin 332 IgG and whether the complement system has a nonobligatory role in the initiation of the inflammatory response (103, 104).

## Anti-laminin 332 reactivity in other pemphigoid diseases

Outside MMP, antibodies against laminin 332 have been detected in individual patients with bullous pemphigoid, anti-p200 pemphigoid, and epidermolysis bullosa acquisita in addition to the autoantibodies against BP180, p200 protein, and type VII collagen, respectively (6–11). The report of anti-laminin 332 reactivity in about 40% of bullous pemphigoid sera was not confirmed in subsequent studies (38, 55, 58, 59). When Holtsche et al. investigated the specificities of serum autoantibodies in anti-p200 pemphigoid, anti-laminin 332 IgG was observed in 43 (18%) of 239 patients in addition to reactivity against the p200 protein and/or laminin γ1 (10).

Autoantibody reactivity in the very rare entity orf-induced pemphigoid has puzzled investigators for many years. Recently, Yilmaz et al, showed that the major target antigen in orf-induced pemphigoid is laminin 332 (5). Of note, while a single patient with orf-induced MMP has been described, all other cases associated with orf did not show predominant mucosal involvement and, consequently may be termed orf-induced pemphigoid when antibodies against laminin 332 are detected or orf-induced epidermolysis bullosa acquisita in case of type VII collagen-specific antibodies (5, 105, 106). The reason why autoimmunity against laminin 332 is not associated with predominant mucosal manifestations when induced by an orf infection is enigmatic. It may be speculated that an underlying molecular mimicry between an orf virus protein and laminin 332 leads to autoantibodies against distinct epitopes on laminin 332 different from those targeted in anti-laminin 332 MMP. Of note, autoantibodies in orf-induced pemphigoid are predominantly of the IgG2 and IgG3 subclasses compared to IgG4 in anti-laminin 332 MMP (38, 107).

## Conclusion

After diagnosis of MMP, testing for serum antibodies against laminin 332 and, when present, a search for the most prevalent solid tumors including chest, abdominal, and pelvic CT, gastroscopy, coloscopy, as well as urological and gynecological examinations appears to be mandatory. The anti-laminin α3 mouse model of MMP may be helpful to decipher key molecules and pathways in the pathophysiology of MMP. Only after definite preclinical data have been generated a randomized controlled treatment study will be initiated and open new therapeutic avenues for patients with this rare and frequently detrimental disorder.

## Author contributions

Supervision: ES. Visualization: ES, SP. Writing - Review and Editing: ES, SP. All authors contributed to the article and approved the submitted version.
